# Comparative analysis of the *LEA* gene family in seven *Ipomoea* species, focuses on sweet potato (*Ipomoea batatas* L.)

**DOI:** 10.1186/s12870-024-05981-x

**Published:** 2024-12-26

**Authors:** Mengqin Hu, Zhenqin Li, Xiongjian Lin, Binquan Tang, Meng Xing, Hongbo Zhu

**Affiliations:** College of Coastal Agricultural Sciences, Guangdong Ocean University, Zhanjiang, Guangdong 524088 China

**Keywords:** *Ipomoea* species, *LEA* gene family, Gene expression, Abiotic stress, Sweet potato

## Abstract

Late Embryogenesis Abundant (LEA) proteins are extensively distributed among higher plants and are crucial for regulating growth, development, and abiotic stress resistance. However, comprehensive data regarding the *LEA* gene family in *Ipomoea* species remains limited. In this study, we conducted a genome-wide comparative analysis across seven *Ipomoea* species, including sweet potato (*I. batatas*), *I. trifida*, *I. triloba*, *I. nil*, *I. purpurea*, *I. cairica*, and *I. aquatica*, identifying 73, 64, 77, 62, 70, 70, and 74 *LEA* genes, respectively. The *LEA* genes were divided into eight subgroups: LEA_1, LEA_2, LEA_3, LEA_4, LEA_5, LEA_6, SMP, and Dehydrin according to the classification of the *LEA* family in Arabidopsis. Gene structure and protein motif analyses revealed that genes within the same phylogenetic group exhibited comparable exon/intron structures and motif patterns. The distribution of *LEA* genes across chromosomes varied among the different *Ipomoea* species. Duplication analysis indicated that segmental and tandem duplications significantly contributed to the expansion of the *LEA* gene family, with segmental duplications being the predominant mechanism. The analysis of the non-synonymous (Ka) to synonymous (Ks) ratio (Ka/Ks) indicated that the duplicated *Ipomoea LEA* genes predominantly underwent purifying selection. Extensive cis-regulatory elements associated with stress responses were identified in the promoters of *LEA* genes. Expression analysis revealed that the *LEA* gene exhibited widespread expression across diverse tissues and showed responsive modulation to various abiotic stressors. Furthermore, we selected 15 *LEA* genes from sweet potatoes for RT-qPCR analysis, demonstrating that five genes responded to salt stress in roots, while three genes were responsive to drought stress in leaves. Additionally, expression changes of seven genes varied at different stages of sweet potato tuber development. These findings enhanced our understanding of the evolutionary dynamics of *LEA* genes within the *Ipomoea* genome and may inform future molecular breeding strategies for sweet potatoes.

**Supplementary Information:**

The online version contains supplementary material available at 10.1186/s12870-024-05981-x.

## Introduction

Abiotic stressors, such as drought, salinity, and extreme weather, disrupt normal crop growth and development, leading to considerable yield losses and quality degradation [1, 2]. Through ongoing evolutionary processes, plants have developed various mechanistic molecular, physiological, and biochemical adaptations to enhance their resistance to these abiotic challenges [3]. For example, transcription factors (TFs) and protein kinases play crucial roles in regulating downstream signaling pathways that activate and modulate specific stress-responsive genes, resulting in physiological responses such as the synthesis of osmoregulatory compounds [4, 5]. Functional proteins associated with plant stress tolerance, including late embryogenesis abundant (LEA) proteins, are upregulated during late embryogenesis and in nutrient tissues in response to water scarcity. These proteins help eliminate reactive oxygen species (ROS) from cells, thus protecting macromolecules and mitigating damage caused by abiotic stressors [6, 7].

LEA proteins are prevalent across the plant kingdom and serve as osmoprotective agents and facilitators of desiccation damage repair. These glycine-rich proteins, characterized by low molecular weights ranging from 10 to 30 kDa, accumulate significantly during the later stages of embryonic development in response to various abiotic stresses, thereby protecting against extreme environmental conditions, particularly drought stress [8]. Although the classification of LEA proteins varies among different species, they are typically divided into eight groups based on conserved structural domains: LEA_1, LEA_2, LEA_3, LEA_4, LEA_5, LEA_6, Dehydrin, and Seed Maturation Protein (SMP) [9]. Understanding *LEA* gene expression's regulatory mechanisms is essential for advancing modern plant molecular biology. The first LEA protein was identified in *Gossypium hirsutum* in 1981 [10]. With the development of whole-genome sequencing technologies, additional LEA proteins have been characterized in model organisms such as *Arabidopsis thaliana* and *Oryza sativa* [11, 12], as well as in food crops like *Zea mays* and *Triticum aestivum* [13, 14], horticultural crops such as *Cucumis sativus* and *Vitis vinifera* [15–17], and other organisms, including *microbes* and *invertebrates* [18]. While a minority of LEA proteins are localized in the *endoplasmic reticulum* of plant cells, most are found distributed throughout the *nucleus*, *cytoplasm*, and *mitochondria* [19].

The essential role of LEA proteins in plant responses to abiotic stressors has been highlighted in several studies. For example, the overexpression of *AtLEA14* enhanced the expression of salt stress response marker genes in transgenic *A. thaliana*, indicating increased salt tolerance [20]. Similarly, the upregulation of *OsLEA3-2* improved salt and drought tolerance in transgenic *A. thaliana* and *O. sativa* [21]. The *OsLEA* increased rice sensitivity to abscisic acid and enhanced osmotic tolerance under drought conditions [22]. Additionally, elevated changes of *CaLEA1* promoted stomatal closure and activated related downstream gene expression in response to drought and salt stress [23]. Both abiotic stress and signaling molecules induced the *ZmLEA3* expression in leaves, and its overexpression enhanced the osmotic and oxidative stress tolerance in transgenic *Nicotiana tabacum* [24]. Furthermore, other genes implicated in stress tolerance include *TaLEA3* in *Leymus chinensis* [25], *IpLEA* in *Ipomoea pes-caprae* [26], *SmLEA* in *Salvia miltiorrhiza* [27], *SlLEA6* in tomato [28], and *SiLEA14* in *Setaria italica* [29].

The sweet potato (*Ipomoea batatas*), an allohexaploid crop, belongs to the *Convolvulaceae* family within the *Ipomoea* genus, specifically the *Batatas* section. This starchy root vegetable is of considerable global economic significance, serving as a food source, industrial material, and bioenergy resource [30–32]. Two diploid relatives, *Ipomoea trifida* and *Ipomoea triloba*, are employed as model organisms to facilitate sweet potato breeding [33]. Additionally, *Ipomoea nil* and *Ipomoea purpurea* are valuable for research into photoperiodic flowering and flower pigmentation [34]. *Ipomoea cairica*, a perennial vine that blooms year-round, is widely cultivated as an ornamental plant in subtropical and temperate regions and exhibits high changes of bioactive compounds with potential medicinal applications [35]. Furthermore, *Ipomoea aquatica*, commonly known as water spinach, is one of Asia's most beloved leafy vegetables, characterized by aquatic and terrestrial growth forms [36, 37].

This study performed a genome-wide comparative analysis of the *LEA* gene family across seven *Ipomoea* species. A total of 73, 64, 77, 62, 70, 70, and 74 *LEA* genes were identified in sweet potato (*I. batatas*), *I. trifida*, *I. triloba*, *I. nil*, *I. purpurea*, *I. cairica*, and *I. aquatica*, respectively. The identified *LEA* genes underwent phylogenetic analysis, gene structure characterization, chromosomal localization, syntenic analysis, calculation of the Ka to Ks substitution ratio (Ka/Ks), cis-regulatory element (CRE) identification, and expression profiling. Subsequently, 15 differentially expressed genes (DEGs) from sweet potatoes were selected for quantitative reverse transcription PCR (RT-qPCR) analysis. The results revealed that five *IbLEA* genes were responsive to salt stress in roots, while three *IbLEA* genes responded to drought stress in leaves. Additionally, seven *IbLEA* genes exhibited varying expression changes at different sweet potato tuber development stages. These findings provided new insights into the evolution of the *LEA* gene family in *Ipomoea* species and may facilitate future advancements in the molecular breeding of sweet potatoes.

## Material and methods

### Identification of *LEA* genes in *Ipomoea* species

Genomic data for sweet potatoes were sourced from the *Ipomoea* Genome Hub (https://ipomoea-genome.org/). *I. trifida* and *I. triloba* data were retrieved from the Sweetpotato Genomics Resource (http://sweetpotato.plantbiology.msu.edu/). Genomic information for *I. nil* was acquired from the *I. nil* database (http://viewer.shigen.info/asagao/). Data for *I. purpurea* were obtained from the CoGe platform (https://genomevolution.org/coge/GenomeInfo.pl?gid=58735), while genomic data for *I. aquatica* and *I.cairica* came from NCBI (PRJCA002216 and PRJNA820303). To identify candidate members of the *LEA* gene family in *Ipomoea* species, 51 LEA protein sequences from *A. thaliana* (https://www.arabidopsis.org/) were utilized as query sequences for a genome-wide BLASTP analysis across seven *Ipomoea* species [38]. The structural domains of the identified proteins were subsequently analyzed using the Conserved Domain Database (CDD, https://www.ncbi.nlm.nih.gov/Structure/bwrpsb/bwrpsb.cgi), Pfam (http://pfam.xfam.org/), and SMART (http://smart.embl.de/) databases. Candidate genes possessing any LEA_1, LEA_2, LEA_3, LEA_4, LEA_5, LEA_6, SMP, or Dehydrin structural domains were classified as members of the *LEA* gene family.

### Physicochemical properties analysis of *Ipomoea* LEA proteins

The physicochemical properties of all identified LEA proteins in the seven *Ipomoea* species were predicted using ExPASy online tools (http://www.expasy.org/tools/protparam.html). Additionally, the subcellular localization of all identified *LEA* genes in the seven *Ipomoea* species was predicted using the WoLF PSORT online tool (https://wolfpsort.hgc.jp/).

### Phylogenetic analysis of *Ipomoea* LEA proteins

Using IQtree software, we constructed a maximum likelihood phylogenetic tree for *LEA* members across several species, including *A. thaliana*, sweet potato (*I. batatas*), *I. trifida*, *I. triloba*, *I. nil*, *I. purpurea*, *I. cairica*, and *I. aquatica* [39]. The WAG + R3 substitution model was the optimal fit based on the Bayesian Information Criterion (BIC) score, with 1000 ultra-fast bootstrap replicates performed for robustness. The phylogenetic tree was visualized using the iTOL online platform (https://itol.embl.de/login.cgi).

### Chromosome distribution and collinearity analysis of *Ipomoea LEA* genes

Chromosomal localization of all identified *LEA* genes in sweet potato (*I. batatas*), *I. trifida*, *I. triloba*, *I. nil*, *I. purpurea*, *I. cairica*, and *I. aquatica* was performed using TBtools software based on gene annotations [40]. The collinearity relationships of *LEA* genes within species were constructed and visualized using TBtools software.

### Synteny analysis and calculation of Ka/Ks values of *Ipomoea LEA* genes

The synteny analysis in the genomes of the seven *Ipomoea* species was performed and visualized using TBtools software. The nucleotide substitution rates (Ka and Ks) and Ka/Ks ratios of duplicated *LEA* genes were estimated using the TBtools software’s Ka/Ks calculator. Divergence time (T) was calculated as T = Ks/ (2 × 6.5 × 10 ^−9^) × 10 ^−6^ million years ago (Mya).

We classify gene pairs that exhibit 90% similarity and 80% coverage, are positioned on the same chromosome, and are in close physical proximity or adjacent to each other as 'tandem repeat gene pairs.' Conversely, gene pairs that are either distantly positioned on the same chromosome or found on different chromosomes are designated as 'segmental repeat gene pairs.'

### Cis-acting element analysis of the *Ipomoea LEA* genes

The cis-acting element prediction was conducted using 1500 bp sequences upstream of the identified *LEA* genes via PlantCARE (https://bioinformatics.psb.ugent.be/webtools/plantcare/html/). Then, the TBtools software was used to visualize the cis-regulatory element figure.

### Protein conserved domains and motif and gene structure analyses of *Ipomoea LEA* genes

LEA protein sequences were grouped to construct an evolutionary tree while coding regions and genomic sequences were compared to examine gene structure. The proteins' conserved structural domains and motifs were identified using the HMMER search and the Multiple Expectation Maximization for Motif Elicitation (MEME) tool (https://meme-suite.org/meme/tools/meme). All analyses were visualized using TB tools [40].

### Expression pattern analysis of *Ipomoea LEA* genes

Seven dedicated transcriptome data sets have been handpicked to facilitate our investigation into the expression profile of *LEA* genes in the context of sweet potatoes. These include "Xushu 18", which was the subject of plant hormone treatment (PRJNA511028), "Liaohanshu 21" and "Shenshu 28", which were subjected to cold treatment (PRJNA987163) [41], "Xushu 32" and "Ningzishu 1", the focus of potassium treatment (PRJNA1013090) [42], and finally "Guangshu 87" and "Ziluolan" which were used for heat treatment (unpublished). Additionally, "Jishu 26" was selected for its versatility, featuring in various parts of sweet potato-related studies (SRP327312), as well as being used in both salt stress and drought treatment investigations (unpublished) (Table S14).

In addition, the transcriptome data for *I. trifida* and *I. triloba* (Table S14) were obtained from the Sweet Potato Genomics Resource (http://sweetpotato.plantbiology.msu.edu/). The *I. aquatica* dataset, which includes MF (salt-sensitive) and BG (salt-tolerant) variants, was sourced from NCBI BioProject for salt stress (PRJNA1119567).

To better describe our observations, we designate genes as either significantly upregulated or downregulated if they demonstrate a |log2 fold change|> 1.5 relative to CK.

### RT-qPCR analysis of sweet potato *LEA* genes

Our RT-qPCR analysis, based on transcriptome data, examined the expression changes of 15 IbLEA genes, significantly distinguished under conditions of salt and drought stress compared to the control.

This study utilized the sweet potato cultivar "Jishu 26" for RT-qPCR analysis. The plants were cultivated in the experimental field of Guangdong Ocean University, Guangdong, China. For tissue expression analysis, samples were collected from various tissues of 3-month-old "Jishu 26" plants, including flower, flower bud, tender leaf, old leaf, tender stem, old stem, and fibrous root, fibrous root, and tuberous root with a diameter of 4 cm, 8 cm, and 13 cm. For abiotic stress treatments, 25 cm twigs from 3-month-old field-grown "Jishu 26" were cultured in Hoagland solution for 14 days. The twigs were treated in Hoagland solution for salt stress with 0 and 200 mM/L NaCl. They were cultured in Hoagland solution for drought stress with 0 and 300 mM/L mannitol. Fibrous root and leaf samples are collected 0, 3, 6, 9, 12, and 24 h after treatment. Each sample was analyzed in triplicate using Dingfa’s method [43], using the *IbARF* gene as an internal reference, using the primers (Table S4) generated with NCBI. Relative transcript changes were calculated by the 2^−ΔΔCT^ method.

### Protein‑protein interaction (PPI) network construction

Using the default parameters, the online STRING database (https://string-db.org/) was utilized to predict and execute potential protein–protein interaction networks using IbLEA proteins based on known Arabidopsis homologs. Cytoscape (V3.10.2) visualized the resulting network [44].

## Results

### Genome-wide identification and phylogenetic analysis of *Ipomoea LEA* genes

**Fig. 1:**
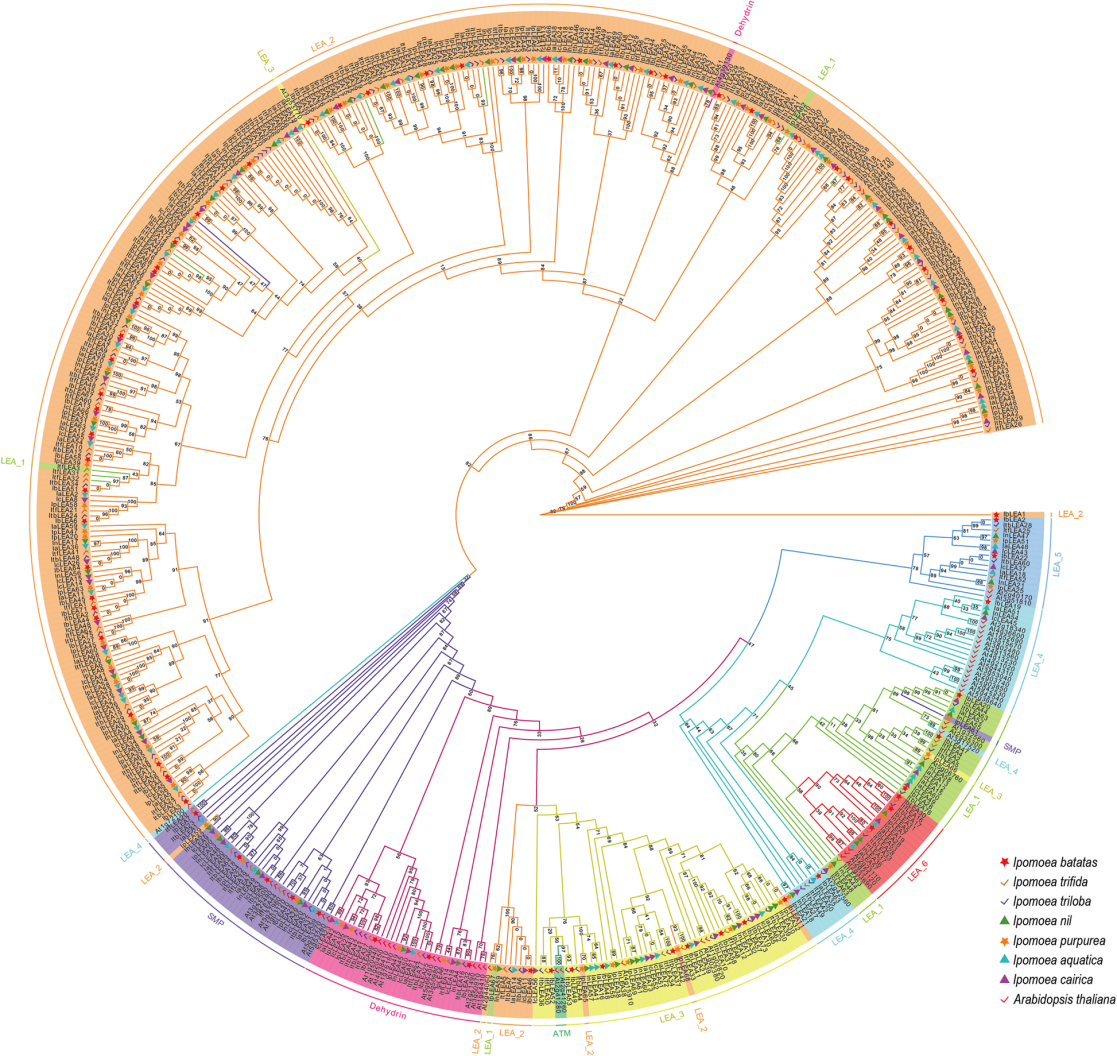
Phylogenetic analysis of *Ipomoea* species and *Arabidopsis* LEA proteins. The proteins were classed into LEA_1, LEA_2, LEA_3, LEA_4, LEA_5, LEA_6, SMP, and Dehydrin, represented by colors such as green, orange, yellow-green, blue, dark blue, red, purple, and pink, respectively. The red star icons, yellow check marks, blue check marks, green triangles, orange stars, blue triangles, purple triangles, and red check marks indicated *IbLEAs* in sweet potato, *I. trifida*, *I. triloba*, *I. nil*, *I. purpurea*, *I. aquatica*, *I. cairica,* and *A. thaliana,* respectively

Sweet potato (*I. batatas*) had 73 *LEA* genes, while its two closely related wild species, *I. trifida* and *I. triloba*, contained 64 and 77 *LEA* genes, respectively. 74, 62, 70, and 70 *LEA* genes were identified in *I. aquatica*, *I. nil*, *I. purpurea*, and *I. cairica*, respectively (Table S1). Furthermore, they all were renamed based on their positions on the chromosomes. A maximum likelihood (ML) tree was reconstructed for the *LEA* members of Arabidopsis and seven *Ipomoea* species. All the 541 *LEA* genes were grouped into eight high bootstrap value (1000) categories based on their corresponding unique structural domains: LEA_1, LEA_2, LEA_3, LEA_4, LEA_5, LEA_6, SMP, and Dehydrin (Fig. 1). The LEA_2 was the largest subgroup including 327 *LEA* genes from *Ipomoea* species, namely, 47 *IbLEA*, 44 *ItfLEA*, 52 *ItbLEA*, 51 *IaLEA*, 39 *InLEA*, 49 *IpLEA*, and 45 *IcLEA*. The LEA_3 was the next largest subgroup, comprising 47 *LEA* from *Ipomoea* species. The remaining.

*LEA* genes from the *Ipomoea* species were divided into other groups: 29 in the SMP, 24 in the LEA_1, 23 in the Dehydrin, and 13, 14 in the LEA_5 and the LEA_6, with 12 in the LEA_4. In sweet potato, there were 47 *IbLEA* genes in the LEA_2 with the most, while 2 *IbLEA* in the LEA_4 and the LEA_5, with the least. There was no mixed presence in each identified group, indicating the relatively conservative distribution of *LEA* gene family members from *Ipomoea* species across different groups.

### Physicochemical properties and subcellular localization of the *Ipomoea* LEAs

The *Ipomoea* LEA proteins exhibited a diverse range of lengths, with the shortest proteins (InLEA5, InLEA36, InLEA37) consisting of 77 amino acids and the longest protein (IbLEA65) comprising 1089 amino acids, averaging 216.91 amino acids in length (Table S2). The molecular weights of these proteins varied from 8.18 kDa (InLEA5) to 122.14 kDa (IbLEA65), with an average of 23.84 kDa. The predicted isoelectric points span from 4.18 to 10.63, with an average pI of 8.66. Among the seven *Ipomoea* LEA proteins, 103 were classified as acidic (pI < 7), and 236 exhibited instability index values exceeding 40. 157 LEA proteins displayed positive average hydrophobicity (GRAVY), while the remaining proteins had negative average hydrophobicity.

IaLEA26 demonstrated an average hydrophobicity of 0, indicating predominantly hydrophilic characteristics among the *Ipomoea* LEA proteins. These *LEA* members were distributed across various organelles, including mitochondria, chloroplasts, cytoplasm, nuclei, cytoskeletons, endoplasmic reticulum, vacuoles, cell membranes, Golgi bodies, and extracellular matrices, with a predominant localization in chloroplasts and cytoplasm.

### Chromosome location and duplication analysis of the *Ipomoea LEA* genes

**Fig. 2:**
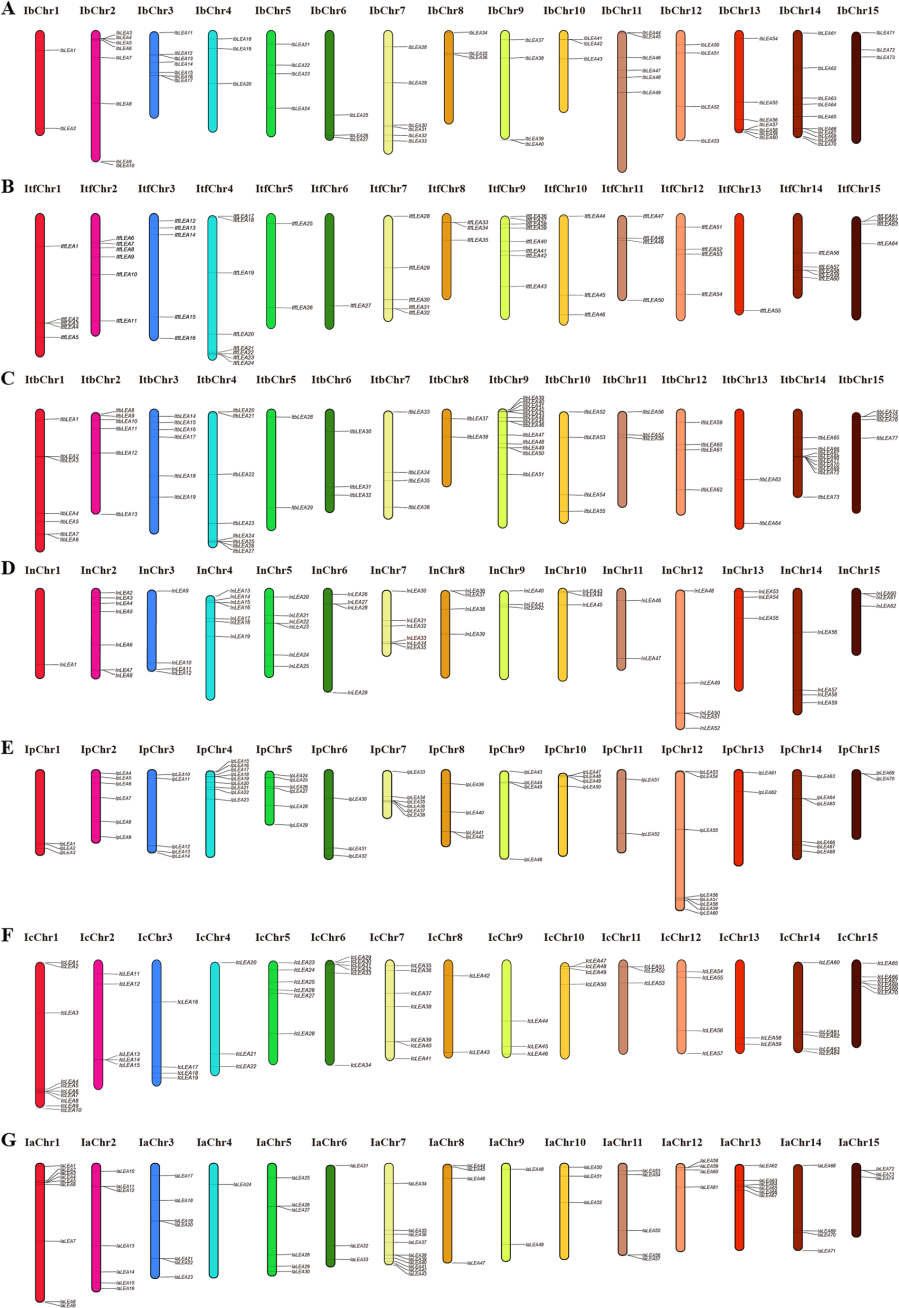
Distribution of *LEA* genes across the chromosomes of the seven *Ipomoea* species. **A** Distribution in sweet potato (*I. batatas*). **B** Distribution in *I. trifida*. **C** Distribution in *I. triloba*. **D** Distribution in *I. nil*. **E** Distribution in *I. purpurea*. **F** Distribution in *I. cairica*. **G** Distribution in *I. aquatica*

In the *Ipomoea* species, the distribution of *LEA* genes across chromosomes was irregular (Fig. 2). The highest number of *LEA* genes occurred 13 across its nine chromosomes in *I. triloba*, whereas the lowest number, 1 *LEA* gene, was present on the ItfChr6 and ItfChr13 in *I. trifida*, the InChr1 of *I. nil,* and the IaChr4 of *I. aquatica*.

**Fig. 3:**
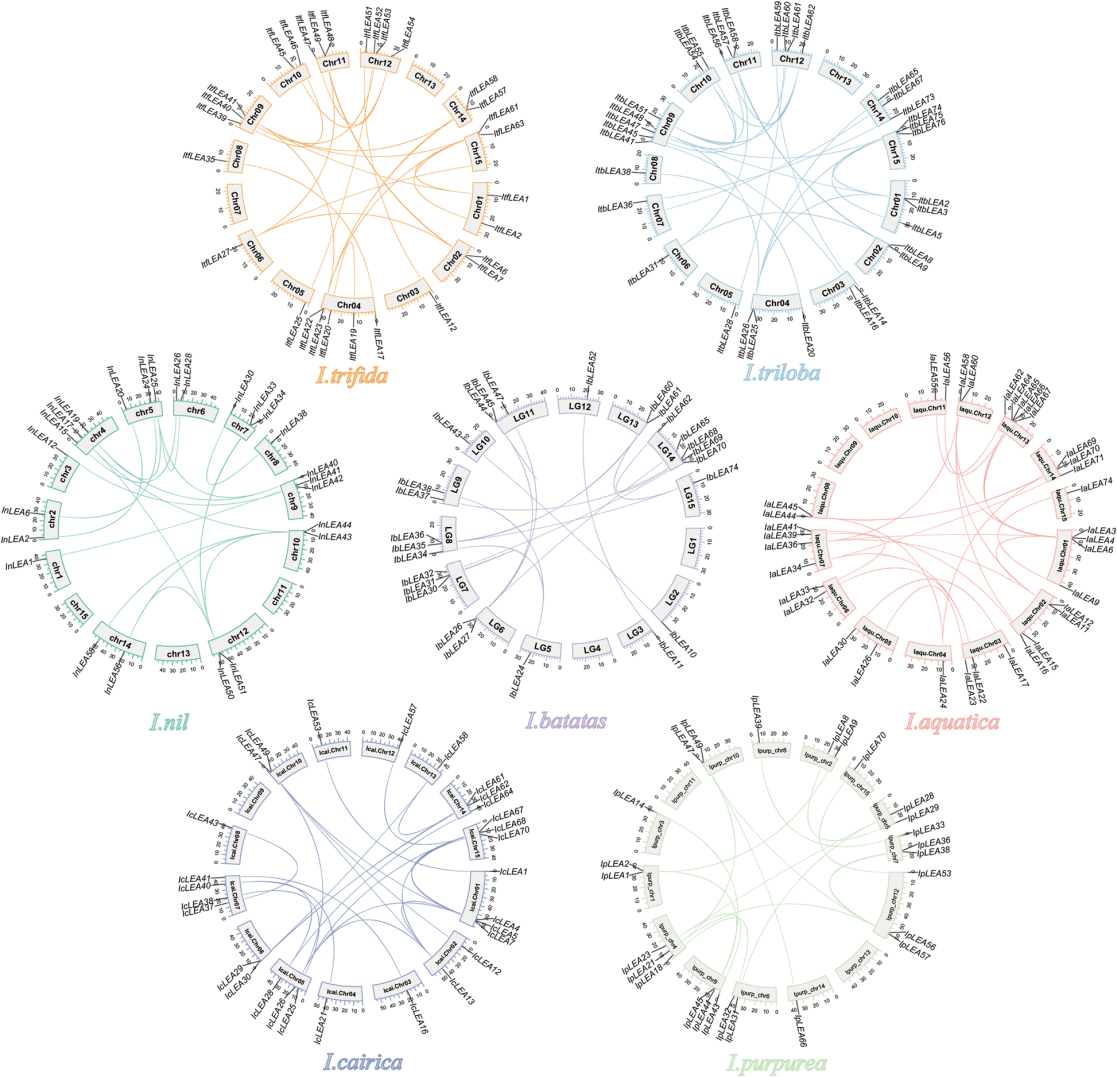
Gene location and collinearity analysis of the *LEA* genes in *Ipomoea* species. The genes were located on different chromosomes. Duplicated gene pairs are linked with a colored line

Collinearity analysis within species revealed the presence of 16 pairs, 21 pairs, 25 pairs, 18 pairs, 16 pairs, and 24 pairs of homologous genes in sweet potato (*I. batatas*), *I. trifida*, *I. triloba*, *I. nil*, *I. purpurea*, *I. cairica*, and *I. aquatica*, respectively (Fig. 3). Notably, only sweet potato and *I. trifida* exhibited tandemly duplicated *LEA* genes, with two pairs and one pair identified, respectively; no tandem duplications were observed in the other five species. Segmental duplication analysis further indicated that sweet potato (*I. batatas*), *I. trifida*, *I. triloba*, *I. nil*, *I. purpurea*, *I. cairica*, and *I. aquatica* contain 14, 20, 25, 18, 16, and 24 pairs of segmental duplicated genes, respectively (Table S3). These results implied that segmental and tandem duplications significantly contributed to the expansion of *LEA* genes in sweet potato and *I. trifida*, with segmental duplication as the predominant mechanism. In contrast, segmental duplication was the exclusive means of *LEA* gene amplification in the other five species.

### Syntenic analysis of *LEA* genes in the genomes of *Ipomoea* species

**Fig. 4:**
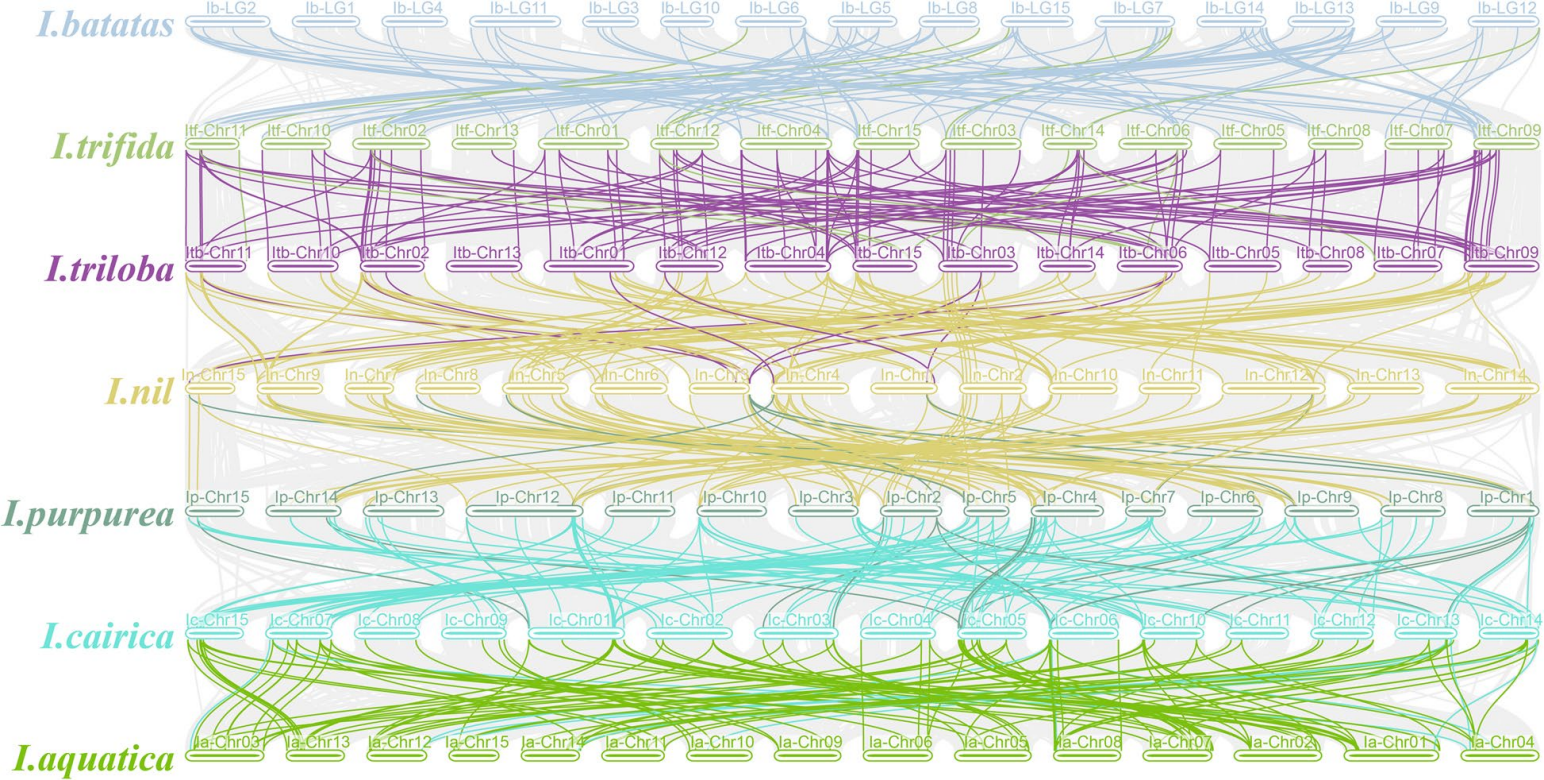
Schematic representation of syntenic genes among sweet potato (*I. batatas*), *I. trifida*, *I. triloba*, *I. nil*, *I. purpurea*, *I. cairica*, and *I. aquatica*. The chromosomes of the seven *Ipomoea* species were reordered. Grey lines in the background indicate the collinear blocks within Ipomoea genomes, with *LEA* gene pairs highlighted in chromatic color

Collinearity results showed that 490 *LEA* genes were detected in these seven *Ipomoea* species, forming 558 orthologous gene pairs (Fig. 4; Table S3). Among them, *I. cairica* and *I.aquatica* had the most orthologous *LEA* gene pairs (108 pairs), followed by *I.purpurea* and *I.cairica* (98 pairs), *I. trifida* and *I. triloba* (98 pairs), sweet potato and *I. trifida* (87 pairs), *I. triloba* and *I. nil* (86 pairs), and *I. nil* and *I.purpurea* (81 pairs).

### Ka/Ks analysis of duplicated and syntenic *Ipomoea LEA* genes

To further investigate the selective pressure on homologous gene pairs in seven *Ipomoea* species, these homologous gene pairs' Ka/Ks values were analyzed (Table S3). Most *LEA* homologous gene pairs' Ka/Ks ratios were less than 1 (95.7%), indicating that these gene pairs were subject to purifying selection during evolution. Six pairs of homologous gene pairs, including.

*ItbLEA16* and *ItbLEA41*, *IpLEA47* and *IpLEA21*, *IpLEA47* and *IpLEA58*, *IaLEA4* and *IaLEA58*, *IcLEA5* and *IcLEA13*, and *IcLEA13* and *IcLEA68*, had Ka/Ks values greater than 1. This suggested that these six pairs of genes underwent positive selection and could have a relatively faster evolutionary rate. There were 16 homologous gene pairs in sweet potato, and the Ka/Ks values of these orthologous gene pairs were all less than 1.

The Ka/Ks analysis of homologous genes among *Ipomoea* species indicates that the occurrence of Ka/Ks ratios exceeding 1 was more prevalent across the seven species than within individual species (Table S3). Specifically, there are 40 pairs (37%) between *I. cairica* and *I. aquatica*, 38 pairs (38.8%) between *I. purpurea* and *I. cairica*, 28 pairs (28.6%) between *I. trifida* and *I. triloba*, 33 pairs (37.9%) between sweet potato and *I. trifida*, 30 pairs (34.9%) between *I. triloba* and *I. nil*, and 32 pairs (39.5%) between *I. nil* and *I. purpurea*.

### Conserved structural domains and gene structure analysis of *Ipomoea LEA* genes

**Fig. 5:**
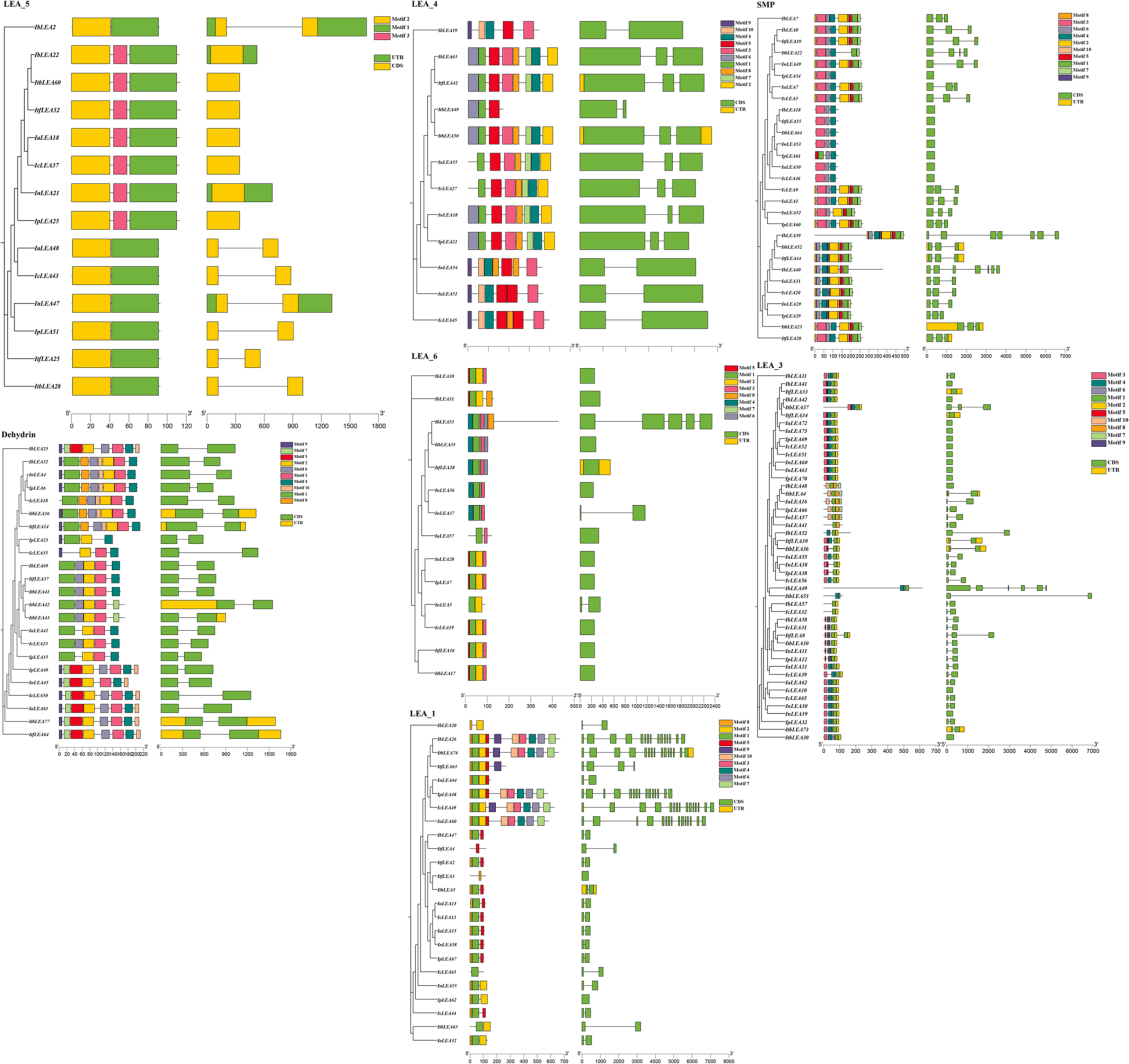
Phylogenetic tree, gene structure and motif compositions of *LEA* genes in *Ipomoea* species. The phylogenetic tree was constructed using IQtree. Different colors represent protein motif analysis, and a number represents each motif

The *LEA* gene family members across seven *Ipomoea* species were categorized into eight distinct subgroups, with phylogenetic trees constructed for each subgroup utilizing maximum likelihood methods (Fig. 5, S1). Closely related *LEA* gene members within the subgroup typically exhibited similar gene structures, particularly regarding intron number and exon length. For instance, LEA_3, 4, 5, 6, SMP, and Dehydrin contain between 0 to 6 introns, with Dehydrin characterized by a single intron. In contrast, LEA_1 and LEA_2 display a broader range of intron counts, with 0 to 14 and 0 to 19 introns, respectively. Notably, LEA_2 exhibits substantial structural variability, housing the highest number of genes and demonstrating significant differences in exon/intron arrangements. These observations implied that functional differentiation within the *LEA* gene family has transpired among the seven *Ipomoea* species.

Distinct motifs were identified within each subgroup, with only three motifs in LEA_5. In comparison, LEA_6 contained eight motifs (Fig. 5). LEA_5 demonstrates a high degree of conservation, encompassing Motif 1 and Motif 2 in all members. In subgroups LEA_1, 3, 4, 6, SMP, and Dehydrin, some genes across different species displayed similarities, and all *LEA* genes within the same species possessed at least one common motif. Conversely, LEA_2 exhibited notable complexity (Fig. S1). This specific classification suggested that *LEAs* within the same subgroup may share analogous biological functions. The variability in conserved sequences indicated that LEA proteins likely evolved through gene amplification events within their respective gene families.

### Cis-regulatory elements in putative promoter regions of the *Ipomoea LEA* genes

Predictive analysis of cis-regulatory elements was performed on the 1500 bp nucleotide sequences upstream of the start codon (ATG) for each *LEA* gene across the seven *Ipomoea* species (Fig. S2). The identified elements included light-responsive cis-regulatory elements associated with abiotic stress or external pressures, plant hormone-responsive cis-regulatory elements, and plant growth and development elements. Light-responsive cis-regulatory elements were the most prevalent, accounting for 4,209 occurrences. Each *LEA* gene within the seven *Ipomoea* species contained these light-responsive elements, with 650 instances identified in sweet potato (46.9%). Six distinct cis-regulatory elements associated with abiotic stress or external pressures were observed, targeting responses to anaerobic conditions, defense and stress, drought, low temperature, MYB factors, and wounding. These results indicated that LEA proteins were likely crucial for drought stress response and tolerance in the seven *Ipomoea* species. The plant hormone-responsive cis-regulatory elements identified included those responsive to abscisic acid, auxin, gibberellin, jasmonic acid, and salicylic acid, suggesting that plant hormones may regulate the expression of LEA proteins. Furthermore, cis-regulatory elements implicated in plant growth and development were also detected, specifically linked to sweet potato meristem expression, endosperm expression, and seed-specific regulation.

### Protein interaction network of *IbLEAs* in Sweet Potato

**Fig. 6:**
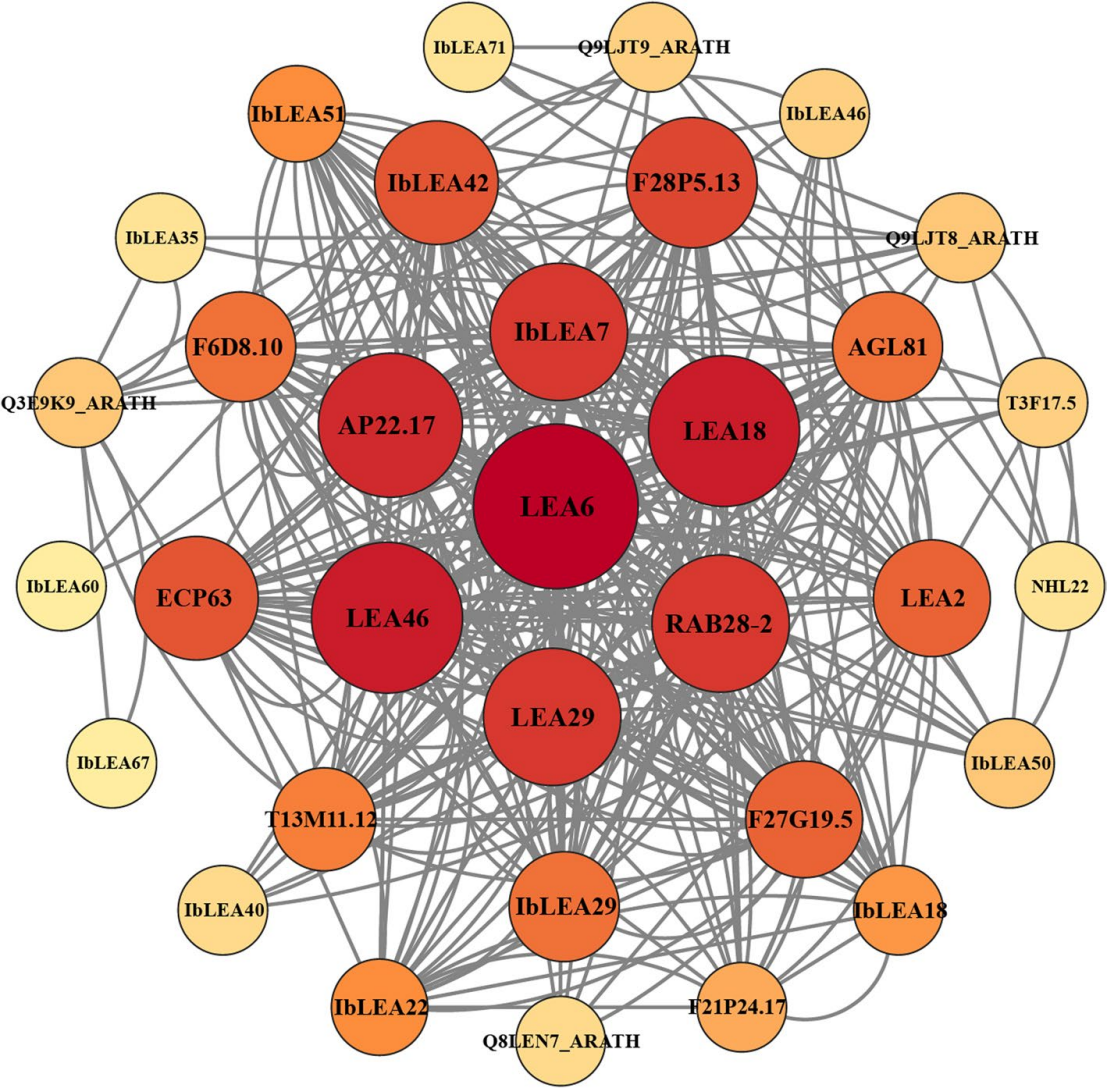
Protein-protein interaction (PPI) network of significant IbLEAs in sweet potato. Nodes represent proteins, central nodes are indicated in red, and black lines indicate interactions between nodes. The darker the color, the more important the protein in the interaction network

An interaction network was generated using homologous proteins from Arabidopsis to investigate the regulatory network of *IbLEAs* (Fig. 6). Interactions among *IbLEAs* were also linked to other proteins. Specifically, *IbLEA42* interacted with *IbLEA60*, *IbLEA18*, *IbLEA46*, *IbLEA50*, *IbLEA22*,

and *IbLEA7*. Furthermore, both *IbLEA42* and *IbLEA7* demonstrated the ability to interact with Glutamate racemase (Q8LEN7). *IbLEA51*, *IbLEA7*, and *IbLEA29* also interacted with the MADS-box protein (AGL81).

### Expression profiles of the *Ipomoea LEA* genes in various tissues

**Fig. 7:**
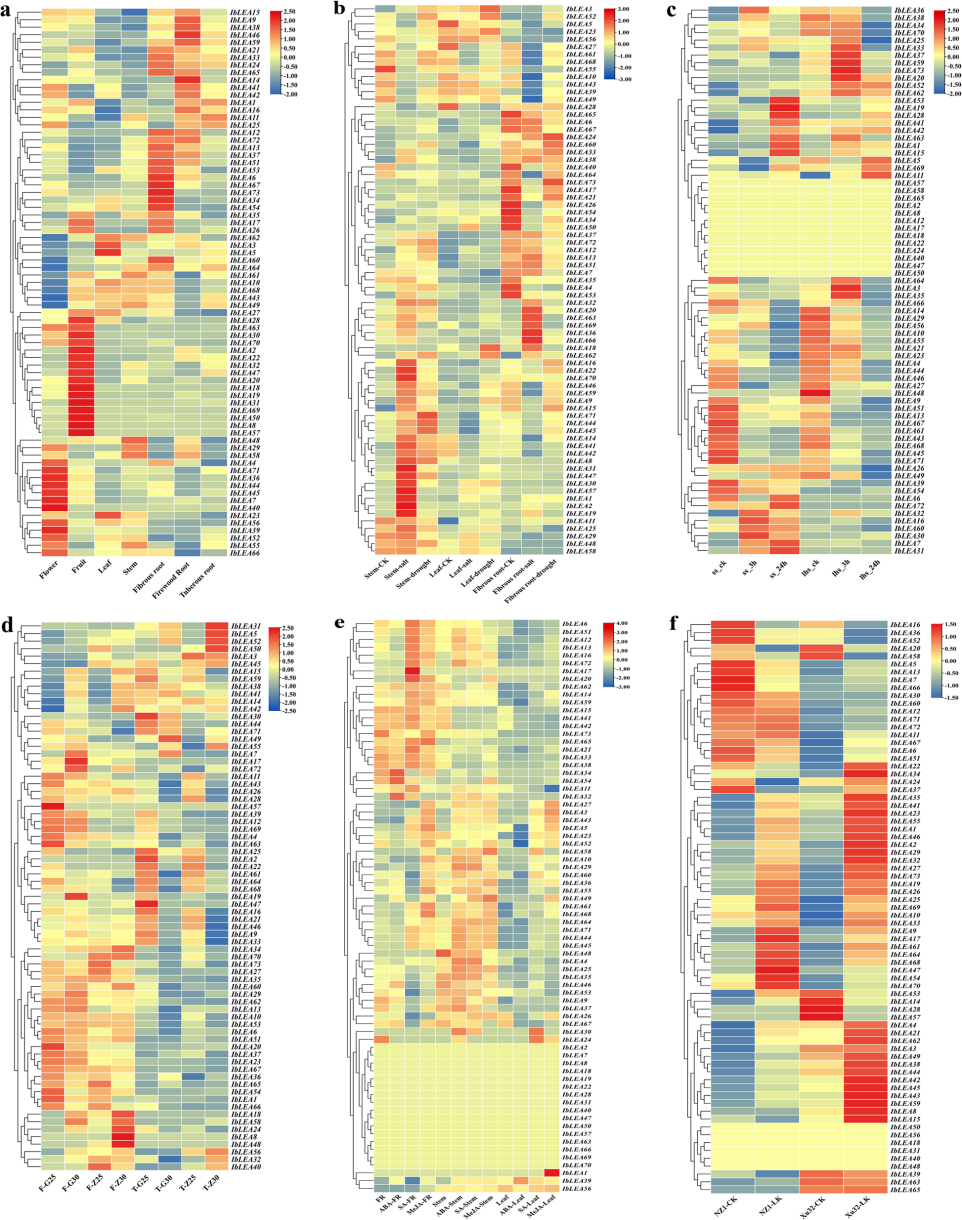
Transcriptome dataset of the *IbLEA* gene across various tissues of sweet potato and in response to abiotic stresses. **a** Expression profiles of the *IbLEA* gene in distinct sweet potato tissues. **b** Expression levels of the *IbLEA* gene in response to salt and drought stress. **c** Expression levels of the *IbLEA* gene under cold stress conditions. **d** Expression levels of the *IbLEA* gene under heat stress conditions. **e** Expression levels of the *IbLEA* gene following hormonal treatments. **f** Expression levels of the *IbLEA* gene under potassium deficiency

The expression profiles of *IbLEA* genes across different tissues of sweet potato were analyzed using transcriptome data, revealing specific expression in flowers, fruits, and various morphological roots (Fig. 7a; Table S9). Notably, eight genes (*IbLEA56*, *IbLEA39*, *IbLEA40*, *IbLEA36*, *IbLEA7*, *IbLEA71*, *IbLEA45* and *IbLEA44*) exhibited exclusive expression in flowers. In comparison, five genes (*IbLEA34*, *IbLEA54*, *IbLEA53*, *IbLEA67*, *IbLEA73*) were uniquely expressed in primary roots. Furthermore, *IbLEA11* showed high expression in tuberous roots, and 16 genes were exclusively expressed in fruits. *IbLEA25*, *IbLEA41*, *IbLEA42*, and *IbLEA15* also demonstrated high expression across multiple sweet potato tissues. We investigated the expression profiles of *LEA* genes across six tissues (flower buds, flowers, stems, leaves, root 1, and root 2) in *I. trifida* and *I. triloba* (Fig. S3a, b; Table S10). In *I. trifida*, two *LEA* genes, *ItfLEA6* and *ItfLEA48*, were exclusively expressed in flowers, while four others exhibit tissue-specific expression in leaves and root 2. Five *ItfLEA* genes were also uniquely expressed in stems (Fig. S3a). In *I. triloba*, five *ItbLEAs* showed elevated expression change expression changes in flowers, and four demonstrated specific expression in root 2 (Fig. S3b).

### Expression profiles of the *Ipomoea LEA* genes under abiotic stress

The expression of *IbLEA* genes under salt and drought stress conditions was examined (Fig. 7b). Significant differences in expression change expression changes were observed for all 73 *IbLEA* genes in tissues subjected to salt or drought stress (Table S9). Under salt stress, eight genes were significantly up-regulated in root tissues, while eighteen were down-regulated. In stem tissues, twenty-two genes were up-regulated, three were down-regulated, whereas in leaf tissues, one was up-regulated, and six were down-regulated. Under drought stress, three genes in root tissues were up-regulated and six down-regulated; in stem tissues, thirteen genes were up-regulated and five down-regulated, while in leaf tissues, eight genes were up-regulated and four down-regulated. These findings indicated distinct expression patterns of *IbLEA* genes were in response to salt and drought stress, with the majority responding to varying stress conditions.

To investigate the genetic resources of *ItfLEAs*, *ItbLEAs*, and *IaLEAs* in response to drought and salt stress, the expression profiles of these *LEAs* were analyzed under these conditions (Table S10). In *I. trifida*, 18 *LEAs* exhibited specific upregulation in response to drought stress, while 15 *LEAs* were upregulated specifically under salt stress (Fig. S3c). In *I. triloba*, 22 *LEAs* were notably upregulated during drought conditions, with 14 *LEAs* showing specific upregulation in response to salt stress (Fig. S3d). In *I. aquatica*, the *LEAs* displayed distinct expression patterns under salt stress in two different salt tolerance varieties. Specifically, after 12 h of salt exposure in the MF (salt-sensitive), the expression of *IaLEA51* increased, whereas *IaLEA55* was upregulated at both the 6-h and 12-h upon salt conditions. In the BG (salt-tolerant), following 6 h of salt exposure, the expression changes of *IaLEA48* and *IaLEA58* increased but subsequently decreased after 12 h (Fig. S3e).

Under high-temperature stress, the expression of the 73 *IbLEA* genes was analyzed (Fig. 7d; Table S8). In the fibrous roots of "Guangshu 87", six genes (*IbLEA38*, *IbLEA41*, *IbLEA42*, *IbLEA14*, *IbLEA15*, *IbLEA59*) were significantly up-regulated. In contrast, in tuberous roots, *IbLEA55* and *IbLEA52* showed significant up-regulation. In the fibrous roots of "Ziluolan," five genes (*IbLEA38*, *IbLEA41*, *IbLEA42*, *IbLEA14*, *IbLEA15*) were similarly up-regulated, with *IbLEA55*, *IbLEA52*, and *IbLEA5* significantly up-regulated in tuberous roots.

Similar analyses were performed for the expression of the 73 *IbLEA* genes under cold stress (Fig. 7c; Table S7). In "Shenshu 28," after three hours of cold stress, *IbLEA15*, *IbLEA41*, and *IbLEA42* were significantly up-regulated, with further increases after 24 h. In "Liaohanshu 21," five genes, including *IbLEA11*, *IbLEA15*, and *IbLEA42*, were significantly up-regulated after three hours, but after 24 h, *IbLEA15* transcript change decreased, and *IbLEA42* and *IbLEA11* expression change increased. Analysis of the expression patterns of *ItfLEAs* and *ItbLEAs* under heat and cold stress (Fig. S3f, g). In *I. trifida*, four *LEAs* were upregulated under heat stress, while twenty-three *LEAs* were upregulated under cold stress. Similarly, in *I. triloba*, eleven *LEAs* were upregulated under heat stress, and twenty-nine *LEAs* were upregulated under cold stress. Further analysis indicated that the differentially expressed *LEA* genes typically responding to abiotic stress primarily belonged in the LEA_2 subgroups (Fig. S4; Table S11).

Using RNA-seq data from "Xushu 18" obtained from the NCBI database (PRJNA511028), the expression patterns of 73 *IbLEA* genes exposed to diverse phytohormone treatments were identified (Fig. 7e; Table S6). In fibrous roots, after ABA treatment, the expression of *IbLEA32*, *IbLEA34*, and *IbLEA54* significantly increased; after MeJA treatment, 15 *IbLEA* genes showed significant up-regulation; and after SA treatment, 18 *IbLEA* genes were significantly up-regulated. In stems, 16 genes were significantly induced by ABA treatment, 15 by SA treatment, and 11 by MeJA treatment. Only *IbLEA39* was significantly up-regulated in the leaves after ABA treatment, whereas 14 genes were up-regulated following MeJA treatment and 12 after SA treatment.

The expression profiles of *ItfLEAs* and *ItbLEAs* were assessed using RNA-seq data from *I. trifida* and *I. triloba* following treatments with GA3, 6-BAP, ABA, and IAA (Fig. S3h, I; Table S10). GA3 treatment specifically upregulated three *ItfLEAs* and eight *ItbLEAs*. In contrast, 6-BAP treatment led to the specific upregulation of twelve *ItfLEAs* and six *ItbLEAs*. Under ABA treatment, twenty-one *ItfLEAs* and twenty *ItbLEAs* showed specific upregulation. No significant alterations in the expression of *ItfLEAs* and *ItbLEAs* were observed following IAA treatment.

Finally, transcriptome data regarding potassium deficiency from the low potassium-tolerant variety "Xushu 32" and the low potassium-sensitive variety "Ningzishu 1" from the NCBI database (PRJNA1013090) were used to analyze the expression of the 73 sweet potato *LEA* genes (Fig. 7f; Table S5). Under low potassium treatment, *IbLEA64* and *IbLEA9* were significantly up-regulated in "Ningzishu 1." In contrast, *IbLEA73*, *IbLEA33*, and *IbLEA32* were significantly up-regulated in "Xushu 32".

### Expression analysis of sweet potato *LEA* genes by RT-qPCR

**Fig. 8:**
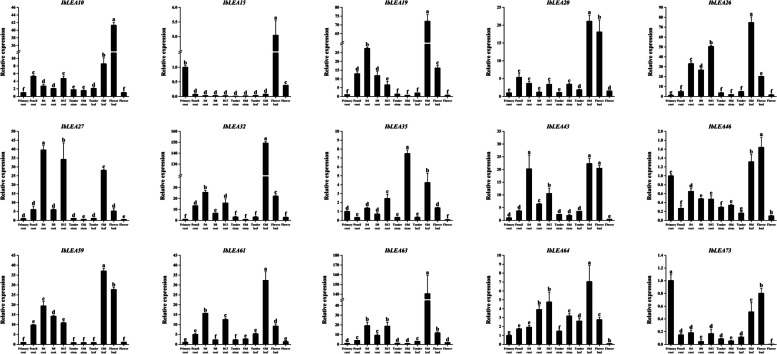
Expression profiles of 15 *IbLEA* genes across various tissues were presented. The x-axis encompasses different tissue types, including primary roots, pencil roots, and tubers of varying sizes (4 cm diameter - DR4, 8 cm diameter - DR8, and 13 cm diameter - DR13), as well as tender stems, old stems, tender leaves, old leaves, flower buds, and flowers. The y-axis denotes the relative expression levels of *IbLEA* genes. Significant differences, indicated by letters a, b, c, etc., are reported at the p < 0.05 level and were determined using one-way ANOVA

Fifteen *IbLEA* genes were selected for RT-qPCR validation. Analysis across various sweet potato tissues, including primary roots, pencil roots, and tubers of varying sizes (4 cm diameter—DR4, 8 cm diameter—DR8, and 13 cm diameter—DR13), tender stems, old stems, tender leaves, old leaves, flower buds, flowers, and tuberous roots at different developmental stages, revealed that all 15 genes exhibited high expression changes in flower buds. Except for *IbLEA15*, the remaining 14 genes have displayed significant expression in old leaves, while *IbLEA35* and *IbLEA64* demonstrated elevated expression in old stems. Notably, five genes (*IbLEA26*, *IbLEA27*, *IbLEA43*, *IbLEA59*, and *IbLEA63*) showed variable expression changes in tuberous roots depending on the growth stage (Fig. 8; Table S12), suggesting they may be potential candidate genes involved in sweet potato tuber development.

**Fig. 9:**
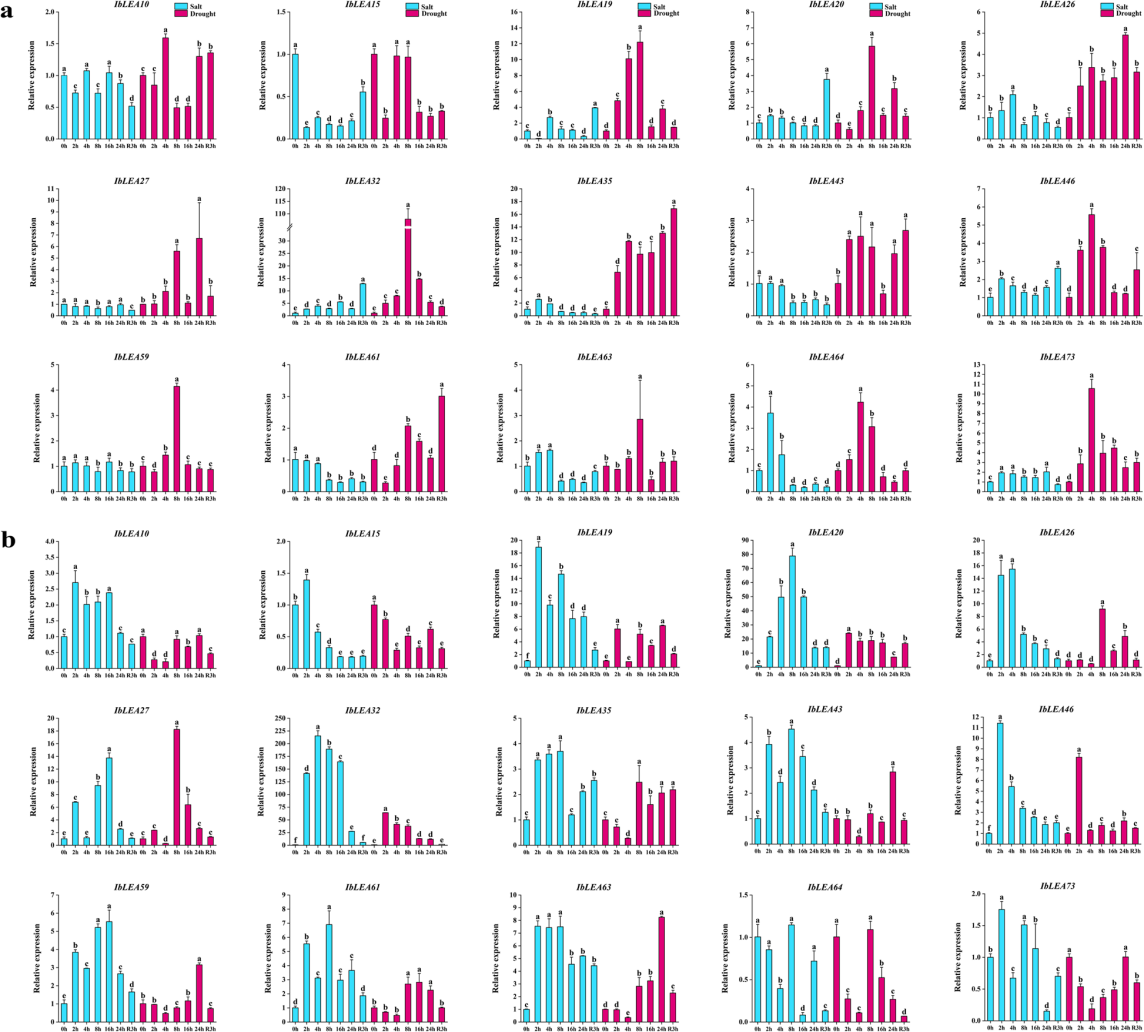
Changes in the expression levels of 15 *IbLEA* genes in different tissues under salt and drought treatments. The letters of a, b, c, etc., indicate significant differences at p < 0.05, as determined by one-way ANOVA with SPSS single-factor tests. **a** Expression of 15 *IbLEA* genes in leaves under salt and drought stress. **b** Expression of 15 *IbLEA* genes in fibrous roots under salt and drought stress

Simultaneously, the expression changes of these 15 *IbLEA* genes were assessed in roots and leaves during re-watering following salt and drought treatments, specifically at 2, 4, 8, 16, and 24 h (Fig. 9; Table S13). The results indicated significant changes in the expression of most *IbLEA* genes across different tissues under both stress conditions. In roots, *IbLEA19* exhibited an 18-fold increase under salt stress at 2 h, followed by a decline from 4 h onward, a trend similarly observed under drought stress. *IbLEA20* began to rise at 2 h under salt stress, peaking at 70 times the control change at 8 h. *IbLEA26* reached 15 times the control change at 4 h under salt stress. *IbLEA27* demonstrated an initial increase followed by a decrease, with a subsequent rise to 13 times the control at 16 h under salt stress and 18 times at 8 h under drought stress. *IbLEA32* consistently increased under both stress conditions, achieving 200 times the control change at 4 h under salt stress and 50 times at 2 h under drought stress. In leaves, under drought stress, *IbLEA19* and *IbLEA20* displayed an initial increase followed by a decrease, reaching 12 times and five times the control changes, respectively, at 8 h, while IbLEA32 showed an overall increase, peaking at 105 times the control change at 8 h.

## Discussion

Sweet potato is a significant tuber crop globally [45]. Two diploid species closely related to hexaploidy sweet potatoes are *I. trifida* and *I. triloba*. Due to their reduced genome sizes and ploidy levels, these species are frequently utilized as model organisms to enhance sweet potato breeding efforts. *I. nil*, an annual climbing herbaceous plant known for its self-pollination and blue flowers, is a model for studying photoperiodic flowering and flower pigmentation [34]. *I. purpurea*, a common agricultural weed with a haploid genome, demonstrates varying changes in herbicide resistance [46]. *I. cairica*, a perennial climbing plant introduced for ornamental horticulture [35]. *I. aquatica*, known as water spinach, is recognized as a popular leafy vegetable with both aquatic and terrestrial traits [36, 37]. The *LEA* gene family is extensively distributed among plants and plays crucial roles in cellular protection against abiotic stress as well as the regulation of growth and developmental processes [6, 47]. The *LEA* gene family has been identified in various crops, including tomato [28], cotton, rice, maize, and wheat [8, 48], and has also been observed in other organisms such as invertebrates and microbes [49]. However, no literature currently addresses the LEA protein family in *Ipomoea* species.

In this study, we identified 73, 64, 77, 62, 70, 70, and 74 *LEA* genes from sweet potato (*I. batatas*), *I. trifida*, *I. triloba*, *I. nil*, *I. purpurea*, *I. cairica*, *I. aquatica*, respectively, totaling 490 *LEA* genes categorized into eight clusters (Fig. 1; Table S1). Among these, the LEA_2 cluster represented the largest subfamily in sweet potato, a finding consistent with observations in tomato [28], *Sorghum bicolor* [50], and *G. hirsutum* [51]. Conversely, the LEA_4 and LEA_5 clusters contained the fewest members, differing from Brassica napus [52], where LEA_4 had the highest member count. This discrepancy was attributed to the compositional variations of *LEA* subfamilies across species. Most identified *LEA* members were localized in the chloroplast and cytoplasm in the seven *Ipomoea* species studied. At the same time, in sweet potatoes, a significant number were found in the chloroplast and cell membrane (Table S2).

Chromosomal localization analyses revealed that *LEA* genes within the *Ipomoea* genus exhibit uneven distribution across the chromosomes of different species (Fig. 2). Such uneven distribution of *LEA* genes has also been observed in other species, including *Juglans regia* and its wild relative *Juglans mandshurica* [53], as well as in rice [11]. Nevertheless, certain gene clusters demonstrated high similarity and collinearity (Fig. 3). The intraspecific collinearity analysis identified 16 pairs, 21 pairs, 25 pairs, 18 pairs, 16 pairs, and 24 pairs of homologous genes in *I. batatas*, *I. trifida*, *I. triloba*, *I. nil*, *I. purpurea*, *I. cairica*, and *I. aquatica*, respectively. Gene duplication events, including segmental and tandem duplications, are critical for the expansion and distribution of plant gene families [54–56]. Our findings indicated that segmental and tandem duplications facilitated the expansion of *LEA* genes in the seven *Ipomoea* species, with segmental duplications being the predominant mechanism (Table S3). This predominance of segmental duplication as a mechanism for *LEA* gene expansion aligns with findings in tomato [28], but differs from those in *A. thaliana* [12, 57], another dicotyledonous plant.

Members of the same LEA protein subgroup typically exhibit one or more conserved structural domains and motifs (Fig. 5, S1), indicating their potential involvement in this group's major and specific functional roles. These conserved domains are integral to the physiological functions of proteins, forming the structural basis for their activities. However, notable structural variances across different subgroups highlight the functional complexity of LEA proteins in *Ipomoea* species. The distribution of conserved motifs implied that genes sharing identical motifs likely originated from expansions within the same group or taxon. Consequently, since different conserved motifs characterized distinct clusters, it can be preliminarily inferred that they correspond to diverse ancestral lineages with unique conserved motifs [17].

The Ka/Ks analysis of duplicated and homologous *LEA* genes within and across seven species of *Ipomoea* revealed that the majority underwent purifying selection during speciation. In contrast, the genes experiencing positive selection were comparatively few (Table S3, S4). Notably, instances of positive selection were more prevalent among homologous *LEA* genes across *Ipomoea* species. These findings indicated that *LEA* genes within *Ipomoea* have predominantly undergone strong purifying selection throughout evolution, resulting in minimal changes post-duplication. At the same time, functional divergences may arise among *LEA* genes across different species.

Cis-regulatory elements in gene promoters are crucial for transcription initiation and regulation [58, 59]. In this study, we conducted a cis-regulatory element analysis on the 1.5 kb upstream promoter region of *Ipomoea LEA* genes. Various CREs associated with plant growth, development, and stress responses were identified (Fig. S2). The identified regulatory elements in *Ipomoea* species include light-responsive cis-regulatory elements, those related to abiotic stress and external pressure, plant hormone-responsive elements, and elements associated with growth and development. Notably, light-responsive cis-regulatory elements were prevalent within the promoters of *Ipomoea LEA* genes, alongside widespread elements pertinent to abiotic stress responses and plant hormone regulation. This suggests that *LEA* family members in *Ipomoea* species may be involved in various plant hormone responses and play critical roles in mechanisms such as abiotic stress defense, stress response, injury recovery, and anaerobic induction. These findings were consistent with prior research on cis-regulatory elements in *LEA* family members across tomato, *T. aestivum*, and *G. hirsutum* [28, 48, 51]. Additionally, elements associated with meristematic tissue expression were detected in sweet potatoes, indicating a potential link between *LEA* genes and the development of sweet potato tubers. Thus, *LEA* genes in *Ipomoea* species were pivotal for adversity resistance, warranting further in-depth investigation.

The study utilized transcriptomic data and RT-qPCR results from diverse sweet potato tissues (Figs. 7a, 8). A correlation analysis was performed to assess the agreement between transcriptome data and RT-qPCR findings, revealing a strong correspondence between the two datasets (Fig. S5). The study outcomes intimate that the *IbLEA* genes could have an impacting role on the development and growth of diverse segments of sweet potato, encompassing flower buds, leaves, fruits, and tubers across different developmental stages. Intriguingly, genes *IbLEA10*, *IbLEA11*, and *IbLEA19* exhibited high expression changes, particularly in flower buds. *IbLEA27*, *IbLEA32*, and *IbLEA59* demonstrate differential expression profiles throughout the various phases of sweet potato tuber development. The DEGs dataset further reinforces this, highlighting their elevated expression changes specifically in the tubers (Fig. 7a), suggesting potential participation in the expansion and growth processes of the tubers. Additionally, the analysis included transcriptomic data from two closely related wild species, *I. trifida,* and *I. triloba*, revealing differential expression of *LEA* genes across tissues in both species (Fig. S3a, b). Notably, the highest number of differentially expressed genes was observed in the flower buds of *I. trifida* and the roots of *I. triloba*.

This study investigated the stress resistance of sweet potatoes by analyzing six transcriptome datasets associated with abiotic stress, acquiring the expression patterns of *IbLEAs* in each dataset (Fig. 7b-f). For comparative analysis, this study also examined and analyzed the *LEA* genes of *I. trifida*, *I. triloba*, and *I. aquatica* in response to abiotic stress (Fig. S3c-i). In sweet potato, the *IbLEAs* exhibited significant responsiveness to various abiotic stresses, with a particularly marked reaction to salt, drought, cold, and low potassium conditions. In *I. aquatica*, *IaLEA51,* and *IaLEA48* exhibit heightened expression at certain time points during salt stress. *ItfLEAs* and *ItbLEAs* demonstrated comparable expression patterns under abiotic stress, revealing greater sensitivity of *LEA* genes to drought stress relative to salt stress and heightened responsiveness to cold stress instead of heat stress. These results offered valuable genetic resources for sweet potato breeding. Similarly, under GA3, 6-BAP, ABA, and IAA treatments, *ItfLEAs* and *ItbLEAs* displayed analogous expression patterns. Notably, ABA treatment resulted in the upregulation of 21 and 20 genes in *ItfLEAs* and *ItbLEAs*, respectively, while IAA treatment did not significantly alter the expression changes of either gene group.

Previous studies have established the critical role of *LEA* genes across multiple species in conferring resistance to abiotic stress. For instance, in soybeans, overexpression of the *GmDHN9* gene, a member of the Dehydrin subgroup of LEA proteins, enhanced drought tolerance in transgenic Arabidopsis [60]. In tomatoes, silencing of *SlLEA6* diminished drought tolerance [28]. In *Ipomoea pes-caprae*, accumulating IpLEA proteins improved the capacity to clear reactive oxygen species (ROS), enhancing survival and tolerance under abiotic stress [61]. In our study, we utilized RT-qPCR to investigate the spatiotemporal expression patterns of 15 *IbLEA* genes under salt and drought stress to elucidate the specific roles of different *IbLEAs* and attain potential candidate *IbLEA* genes (Fig. 9). The expression of *IbLEA* genes was responsive to salt and drought stress, with certain genes exhibiting rapid induction and sustained high expression during specific time intervals, indicating distinct functional roles in various stress contexts. Notably, *IbLEA* genes in leaves show greater sensitivity to drought stress than high salt stress, whereas in roots, they were more responsive to high salt than drought. Interestingly, some *IbLEA* genes display similar expression patterns in response to salt and drought stress, supporting findings from multiple studies that underscore the significant role of *LEA* genes in mediating drought stress responses [8]. In conclusion, the integration of transcriptome data from sweet potato subjected to salt and drought stress, in conjunction with RT-qPCR analysis of 15 *IbLEA* genes, led to the identification of eight potential candidate genes, *IbLEA32*, *IbLEA64*, *IbLEA27*, *IbLEA59*, *IbLEA26*, *IbLEA20*, *IbLEA15*, and *IbLEA63*, that may be crucial for stress resistance in sweet potato.

## Conclusion

In this study, we comprehensively identified the *LEA* gene family members in sweet potato and six closely related species. We identified 73, 64, 77, 62, 70, 70, and 74 *LEA* genes in sweet potato (*I. batatas*), *I. trifida*, *I. triloba*, *I. nil*, *I. purpurea*, *I. cairica*, and *I. aquatica*. Phylogenetic analysis revealed that *LEA* genes in the genus *Ipomoea* were categorized into eight subgroups, consistent with findings in other plant species. Both phylogenetic and syntenic analyses indicated that the *LEA* gene family was evolutionarily conserved. Additionally, we explored gene structures, motif organizations, duplication events, homology analysis, and the presence of cis-regulatory elements. Analysis of expression profiles revealed that *LEA* displayed tissue-specific and varied expression patterns in sweet potato and its two closely related species. Based on the expression profiles of *IbLEA* genes, we selected 15 DEGs for RT-qPCR analysis under salt and drought stress, indicating that eight *IbLEA* genes may be involved in sweet potato's resistance mechanisms. This study contributed to our understanding of the evolutionary dynamics of *LEA* genes within *Ipomoea* species and provided a foundation for resistance breeding efforts in sweet potatoes.

## Supplementary Information

Supplementary Material 1.

## Data Availability

All datasets supporting the results of this study are included in this article and its Supplementary data files. The data and materials that support the findings of this study are available from the corresponding authors upon reasonable request.
