# Supplementary material for: Comparative analysis of the LEA gene family in seven Ipomoea species, focuses on sweet potato (Ipomoea batatas L.)
Source: BMC Plant Biol. 2024 Dec 26;24:1256. doi: 10.1186/s12870-024-05981-x (PMC11670493; doi:10.1186/s12870-024-05981-x)
Supplement: Supplementary file 1 — Supplementary Material 1. [file 12870_2024_5981_MOESM1_ESM.zip › Supplement Figures.pdf]

LEA_2

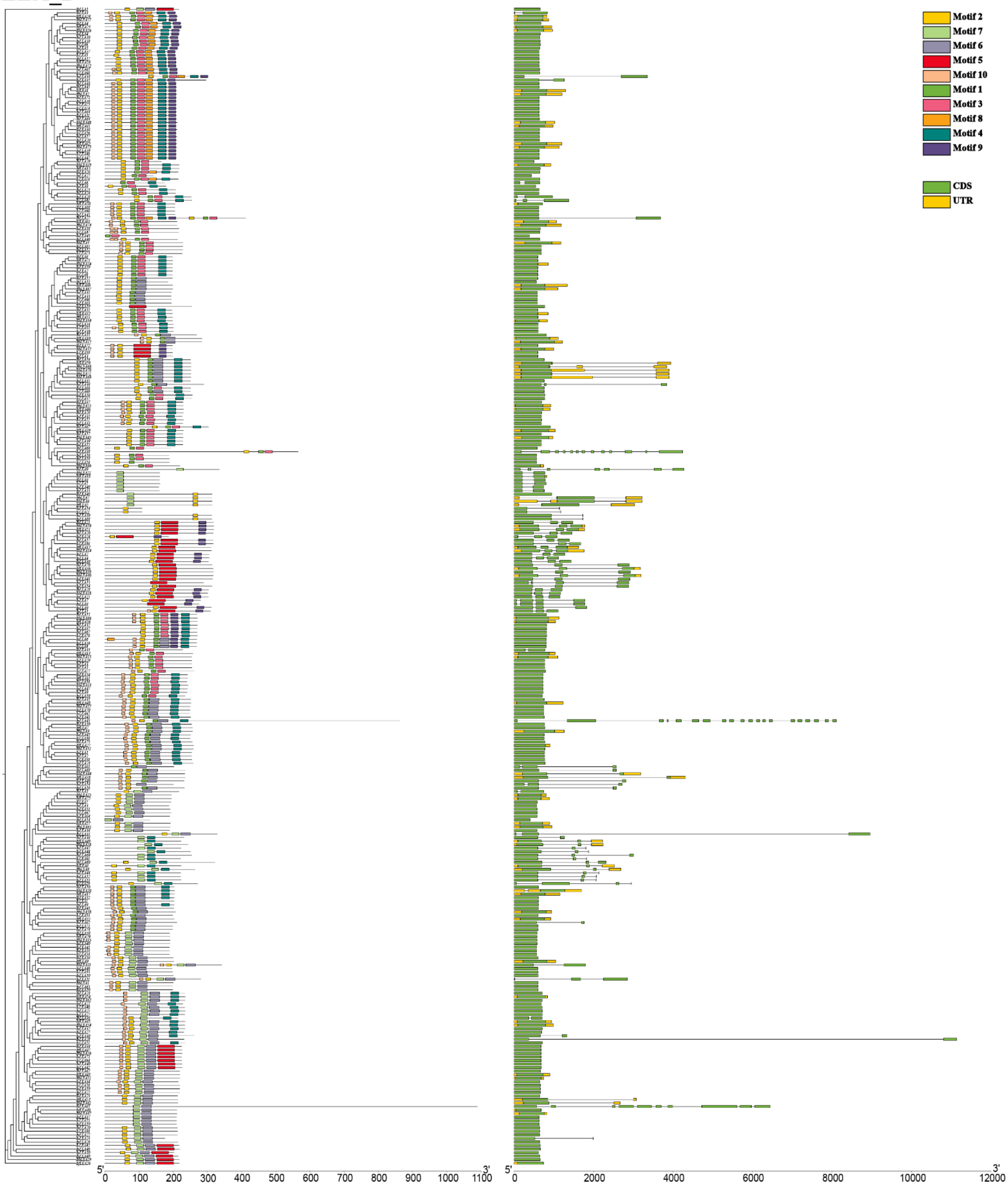

Figure. S1 Phylogenetic tree, gene structure and motif compositions of *LEA* genes in *Ipomoea* species. The phylogenetic tree was constructed using IQ tree. Protein motif analysis is represented by different colors, and each motif is represented by a number.

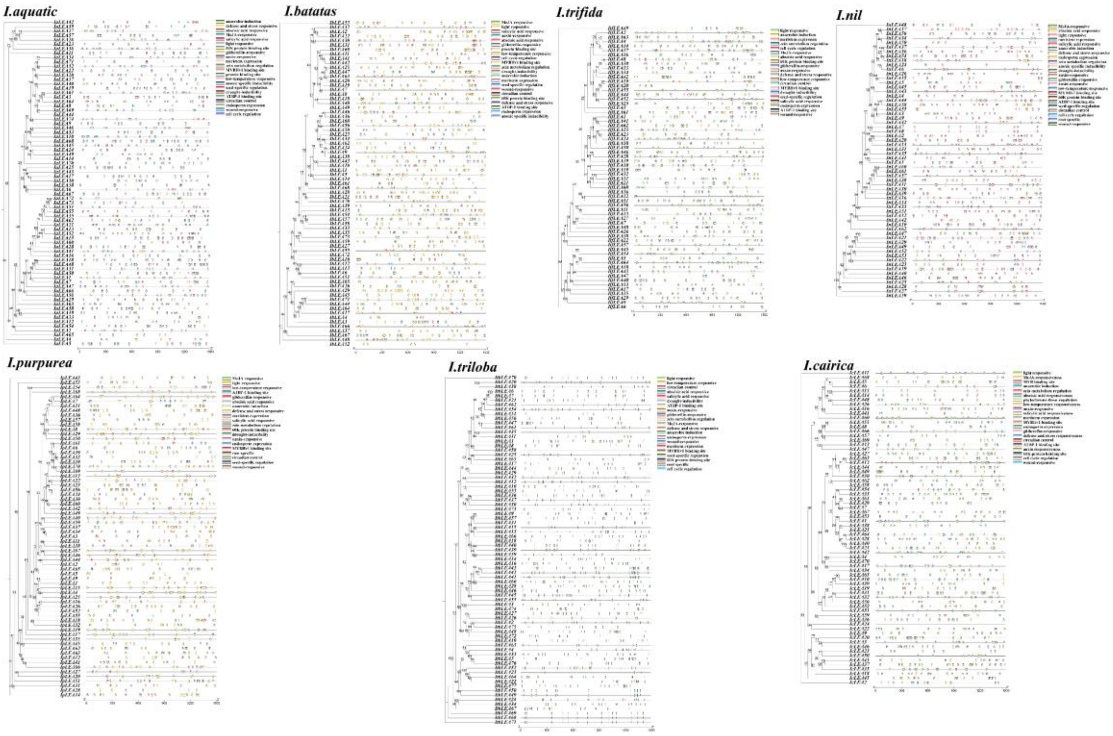

Figure. S2 Distribution of cis-acting elements of the *LEA* gene family in *Ipomoea* species. Distribution of cis-acting elements identifed in the 1500 bp upstream promoter region of the *Ipomoea LEA* gene.

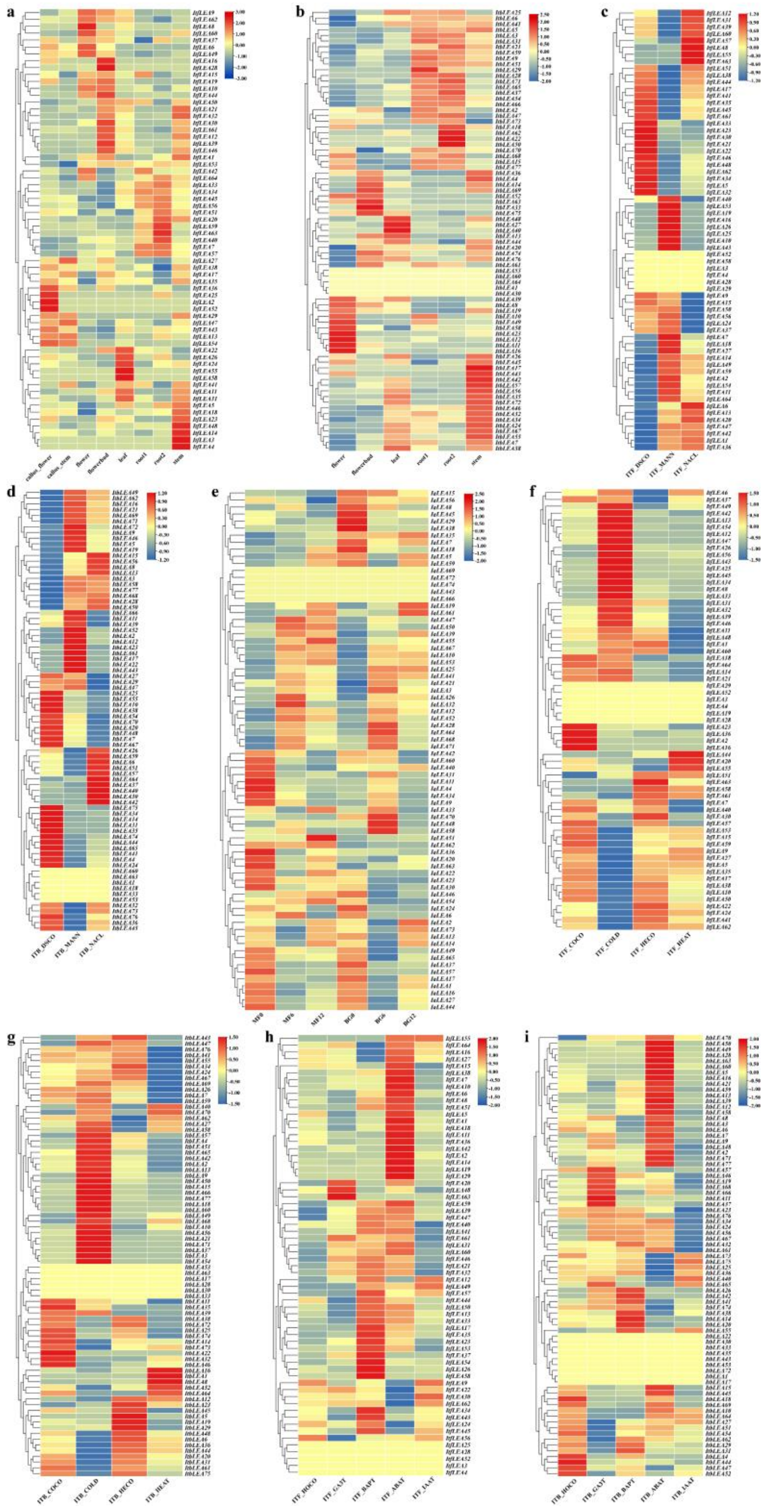

Figure S3 The transcriptome datasets of *LEA* genes in different tissues and their responses to abiotic stress. (a) Expression profiles of the *ItfLEA* gene in distinct *I. trifida* tissues. (b) Expression profiles of the *ItbLEA* gene in distinct *I. triloba* tissues. (c) Expression levels of the *ItfLEA* gene in response to salt and drought stress. (d) Expression levels of the *ItbLEA* gene in response to salt and drought stress. (e) Expression levels of the *IaLEA* gene in response to salt and drought stress. (f) Expression levels of the *ItfLEA* gene under heat stress conditions. (g) Expression levels of the *ItbLEA* gene under heat stress conditions. (h) Expression levels of the *ItfLEA* gene following hormonal treatments. (i) Expression levels of the *ItbLEA* gene following hormonal treatments.

	Salt				Drought			Cold			Heat		
	I. batatas	*I. trifida*	*I. triloba*	*I. aquatica*	*I. batatas*	*I. trifida*	*I. triloba*	*I. batatas*	*I. trifida*	*I. triloba*	*I. batatas*	*I. trifida*	*I. triloba*
LEA_1	3	0	0	0	1	0	0	0	0	0	0	0	0
LEA_2	21	7	10	1	8	8	10	11	18	11	6	7	15
LEA_3	3	2	1	0	1	1	0	4	1	2	2	1	1
LEA_4	2	0	2	0	0	0	1	0	0	1	0	1	0
LEA_5	1	0	0	0	0	1	0	0	0	0	0	0	0
LEA_6	0	0	0	1	1	0	0	1	0	0	0	0	0
SMP	0	0	1	0	0	0	2	0	1	1	0	1	1
Dehyrin	2	1	1	1	2	0	0	1	0	0	0	1	0

Figure S4 Comparison of subfamily-based gene numbers under abiotic stresses in the sweet potato, *I. trifida*, *I. triloba*, and *I. aquatica*.

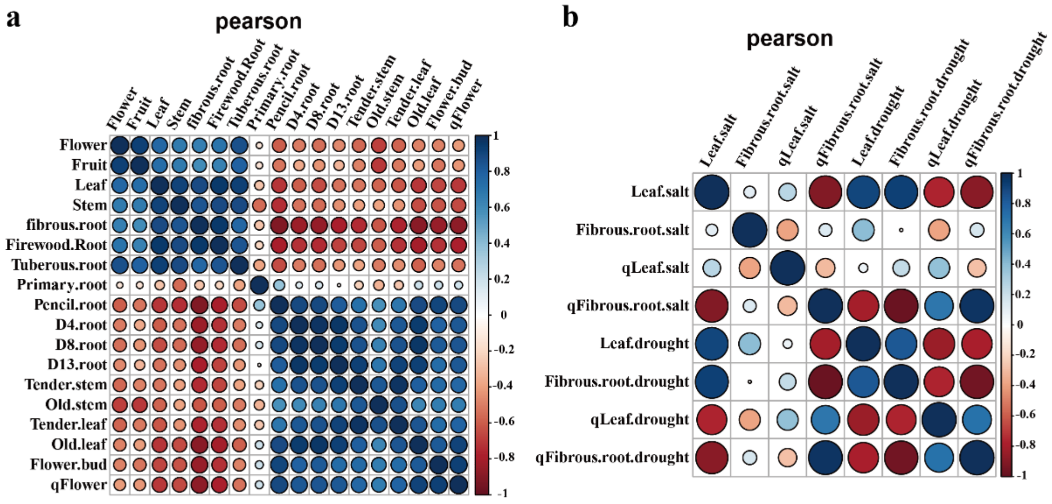

Figure S5 Pearson Correlation Analysis. (a) Analysis of consistency between transcriptome data and RT-qPCR results of sweet potato tissue. (b) Analysis of consistency between transcriptome data and RT-qPCR results of sweet potato under salt and drought stress.
