# Supplementary figures and images for: Comparative analysis of the LEA gene family in seven Ipomoea species, focuses on sweet potato (Ipomoea batatas L.)
Source: BMC Plant Biol. 2024 Dec 26;24:1256. doi: 10.1186/s12870-024-05981-x (PMC11670493; doi:10.1186/s12870-024-05981-x)

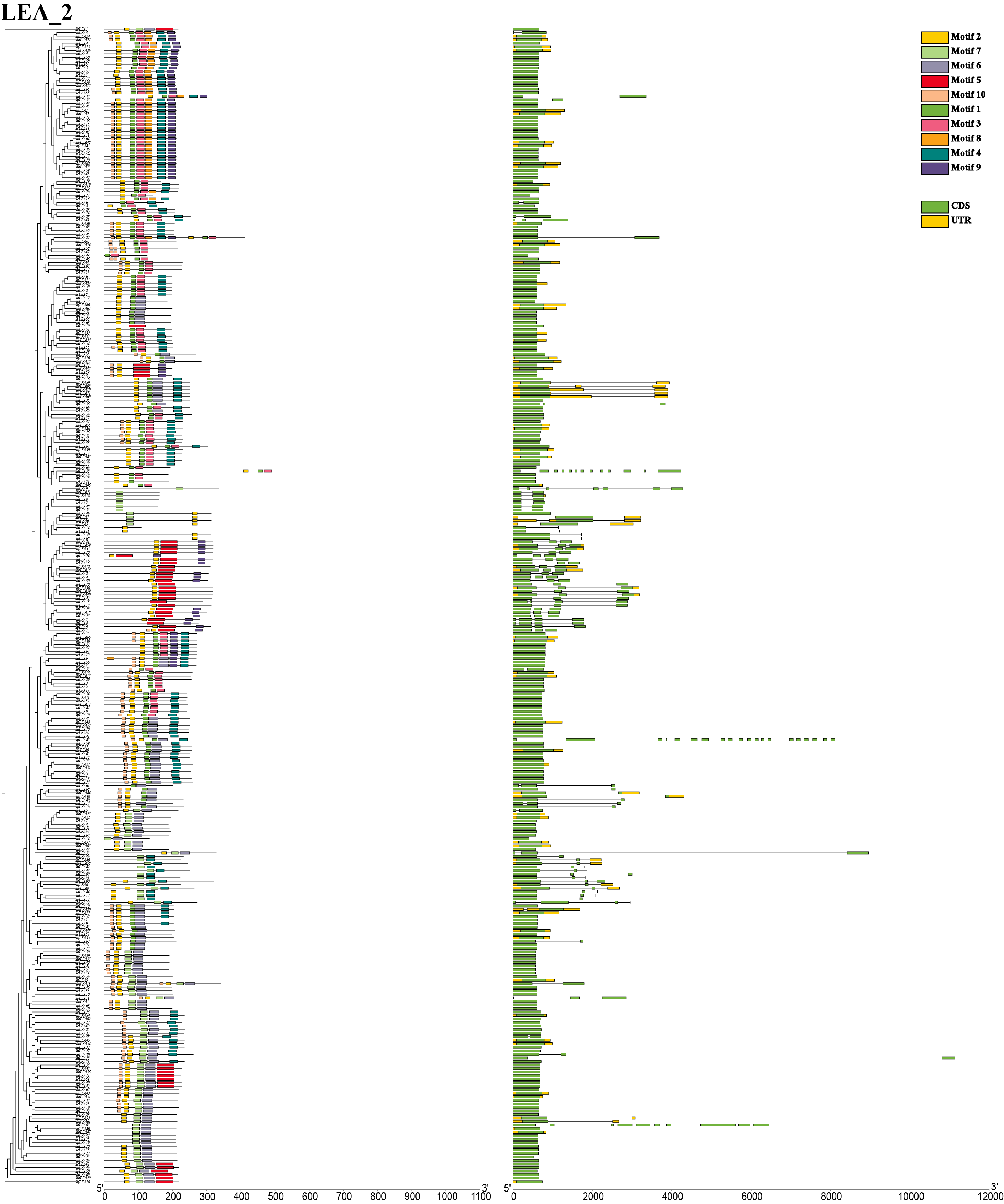

Supplement: Supplementary file 1 — Supplementary Material 1. [file 12870_2024_5981_MOESM1_ESM.zip › Supplement Figures/Fig. S1 Conserved structural domains and gene structure analysis of LEA_2.png]

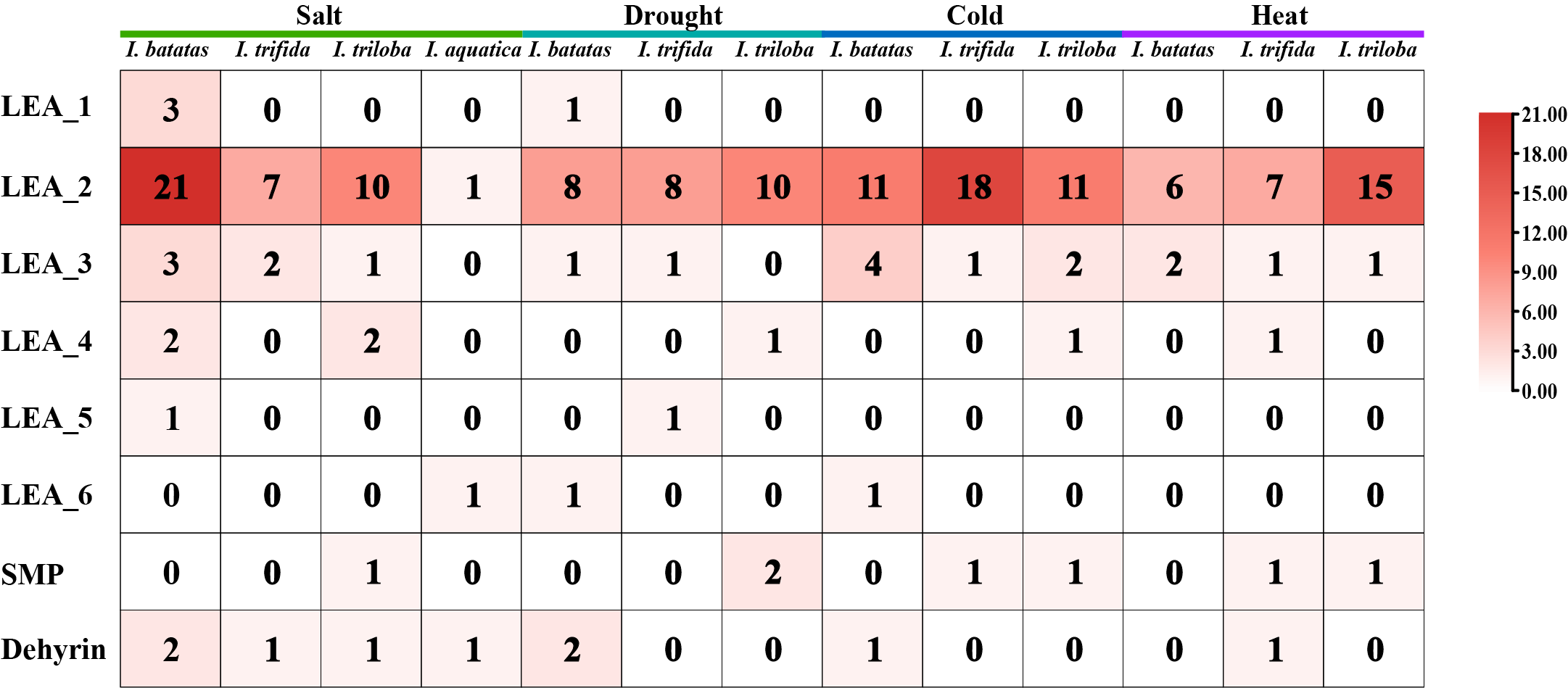

Supplement: Supplementary file 1 — Supplementary Material 1. [file 12870_2024_5981_MOESM1_ESM.zip › Supplement Figures/Fig. S4 Number of DEGs gene.png]

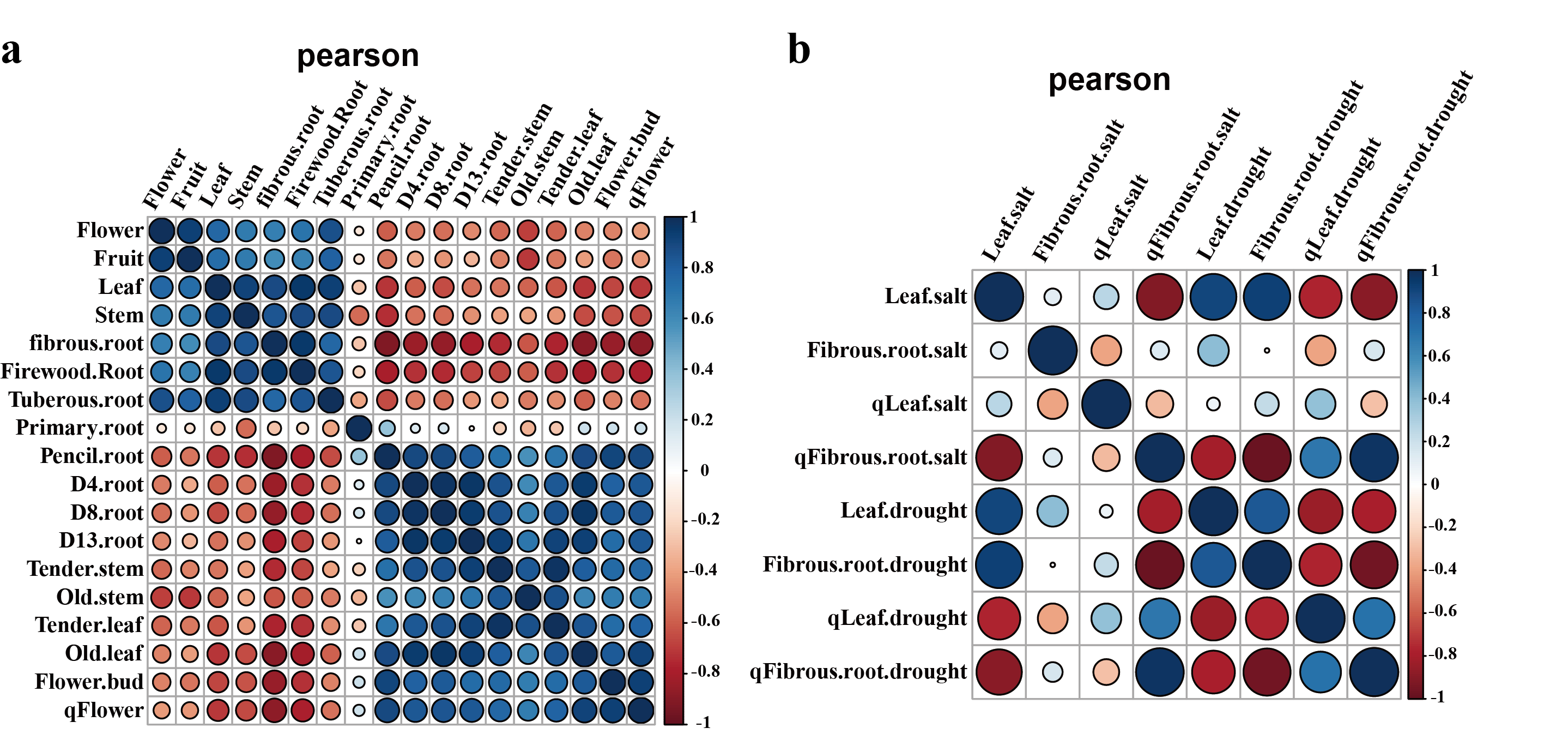

Supplement: Supplementary file 1 — Supplementary Material 1. [file 12870_2024_5981_MOESM1_ESM.zip › Supplement Figures/Fig. S5 pearson.png]
